# Multifactorial influence on duration of exclusive breastfeeding; a Danish cohort study

**DOI:** 10.1371/journal.pone.0238363

**Published:** 2020-09-01

**Authors:** Hanne Kronborg, Else Foverskov

**Affiliations:** 1 Department of Public Health, Aarhus University, Aarhus, Denmark; 2 Department of Public Health, Section of Social Medicine, University of Copenhagen, Copenhagen, Denmark; University of North Carolina at Greensboro, UNITED STATES

## Abstract

The multifactorial aspects of breastfeeding require measures at many levels to identify mothers in need of breastfeeding support from healthcare professionals. Our objective was to examine the relative importance of sociodemographic, pre/perinatal-, infant-, psychosocial-, and interaction-related factors affecting duration of exclusive breastfeeding. We used self-reported data from a community-based trial including 1265 women (response rate 49%) giving birth from January 2017 to February 2018. Data on outcome, duration of exclusive breastfeeding, were collected three and nine months postpartum; data on the study variables concerning known risk factors for breastfeeding cessation were collected two weeks postpartum. Crude and multiple Cox proportional hazards models were used for statistical analyses with additional analyses for time varying effects. Factors with an independent prognostic influence on duration of exclusive breastfeeding in fully adjusted models included early skin-to-skin contact (HR = 1.18 CI:1.04–1.33), intention to breastfeed (HR = 0.77 CI: 0.73–0.80), positive outcome evaluation, meaning the value mothers attributed to breastfeeding (HR = 1.33 CI: 1.08–1.63), higher level of self-efficacy (HR = 1.46 CI: 1.24–1.72), and maternal sense of security in relation to breastfeeding (HR = 1.31 CI: 1.14–1.50). Higher maternal BMI, lower self-efficacy, shorter breastfeeding duration of previous child, and hospitalization during birth were time dependent by affecting the exclusive breastfeeding duration primarily in the first months following birth. The results suggest that target groups in special need of early breastfeeding support are defined by being hospitalized, obese, having low self-efficacy or short previous breastfeeding experience. The extensive influence of psychosocial factors emphasizes the importance of including both practical facilitating guidance and positive verbal encouragement to ensure effective breastfeeding support.

## Introduction

Exclusive breastfeeding is the optimal nutritional strategy in the first six months of the infant’s life [1]. Despite the well-documented short- and long-term health benefits for both the infant and the mother, breastfeeding duration is still shorter than World Health Organization recommendations in both developing and developed countries [2]. During the last decade, research has repeatedly shown that it is possible to increase exclusive breastfeeding rates by increasing supportive practices in health care services [3]. A recent systematic review investigating initiatives to improve breastfeeding practices pointed to the multifactorial aspects of breastfeeding and the need of multiple measures to identify the prerequisites for and content of effective breastfeeding support [4].

In longitudinal cohort studies in western countries, a number of both maternal and infant factors have been found to influence the duration of exclusive breastfeeding. Maternal characteristics for early cessation of breastfeeding have been linked to sociodemographic, pre/perinatal, and psychosocial determinants. Sociodemographic maternal factors related to a shorter exclusive breastfeeding duration include lower age [5], lower level of education [5,6], smoking [5,7,8], and increased pre-pregnant body mass index [9,10]. Pre/perinatal factors include first-time motherhood or previous short breastfeeding experience in multiparous women [6,11–13], mode of birth delivery—especially caesarean section [13], absent supportive hospitalization practices and lack of guidance from health professionals [14,15], as well as behavioral factors such as the experience of early breastfeeding problems [13,16,17]. Last, increasing evidence has shown that low levels of psychosocial factors such as intention [18–20], attitudes [8], confidence/self-efficacy [18–20], and perceived social support [19] are associated with early cessation of breastfeeding. Maternal characteristics related to early mother-infant interaction may also play a role. However, findings are contradictory concerning the influence on breastfeeding duration of postnatal depression [21] and early mother-infant interaction [22]. Infant characteristics negatively influencing breastfeeding duration include male sex [23], lower gestational age [24,25], reduced newborn skin-to-skin contact [26], and early pacifier use [8,16].

Previous literature on factors influencing duration of exclusive breastfeeding has only investigated some of the factors in each study. However, studying the combination of known influential factors is important to obtain a whole picture and avoid biased results. We used data from a community-based trial and included all factors known to influence breastfeeding cessation in a multiple analysis to find out which factors health professionals should focus on concerning maternal needs for breastfeeding support. Our objective was to consider the multifactorial structure of breastfeeding and examine the relative importance of sociodemographic, pre/perinatal-, psychosocial-, interactional-, and infant-related factors influencing duration of exclusive breastfeeding when also accounting for the timing of breastfeeding cessation.

## Materials and methods

### Study design and setting

We used a cohort design with data on mothers included in a community-based cluster randomized trial [27]. The study took place in four Danish municipalities, geographically representing both urban and agricultural areas as well as providing a variation in sociodemographic characteristics of the inhabitants. In Denmark, multiparous women giving birth without complications are treated on an outpatient basis. This also applied to first-time mothers in two of the included municipalities, whereas two other municipalities placed in different geographical regions offered two days of hospitalization for first-time mothers. Hospital services, both maternity and postnatal wards as well as neonatal intensive care units (NICUs) follow the Baby Friendly Initiative. Shortly after discharge, a health visitor employed by the municipality routinely visits families with newborns. The content of this home visit is not standardized, but depends on the expressed needs of the mother and infant. The first visit often concerns supporting breastfeeding and parental infant attachment.

### Participants and ethical conditions

All women who were residents in the study area and gave birth between 1 January 2017 and 28 February 2018 were eligible for participation. At the health visitor’s first home visit after birth, new mothers received an invitation to participate in the study. The only exclusion criterion were mothers unable to manage own legal affairs and therefore offered another kind of support than a visit by a health visitor.

The Central Denmark Region Committee on Health Research Ethics confirmed that this study was exempted from notification according to Danish law (ID 172/2016). Participating mothers were informed about the study verbally and in writing before enrolment and provided written informed consent. Permission to retrieve and store data was obtained from The Danish Protection Agency (ID 2015-57-0002).

### Data collection

Data were collected using self-reported questionnaires, mainly distributed online and with a personal login. Printed questionnaires were available for participants with no access to computers. Data collection took place at three time points: at two weeks, at three months and at nine months postpartum [27].

### Measures

The study variables were obtained from the questionnaire filled out at two weeks postpartum and the outcome variable was obtained from the subsequent two questionnaires.

**The outcome variable** was duration of exclusive breastfeeding, defined as a child being fed only on mothers’ milk according to WHO´s indicators for assessing breastfeeding practices [28]. Mothers were asked: How many months and weeks did your child only have breast milk without any other supplement at all? After this, we converted the variable into full weeks and corrected for the time mothers stated in other questions they had started to give food other than breast milk.

**The study variables** were allocated into groups reflecting sociodemographic, pre/perinatal-, infant-, mother-infant interaction related, and psychosocial factors presented in the following.

**Sociodemographic factors** included questions on age, educational level measured as completed vocational education, pre-pregnancy body mass index and smoking.

**Pre/perinatal factors** included questions on parity, duration of breastfeeding last child measured as none for first-time mothers, 0–5, 6–17, and >17 week for multiparous women, data on delivery and hospitalization measured as homebirth, treated on an outpatient basis or hospitalized more than 24 hours following birth. Early information from health professionals included answers to five questions concerning maternal perception of information about taking care of the infant, breastfeeding, infant's cues, sleep, and crying. Responses were given on a five-point Likert scale and subsequently summed to a scale with a range of 0–25. Early physical breastfeeding problems experienced in the first two weeks following birth were addressed by six questions concerning painful breastfeeding, difficulty latching on, sore or injured nipples, milk stasis, mastitis, and insufficient infant weight gain. Mothers answered whether they had a problem in week one and/or week two and all answers were summed to a scale ranging between 0 and 12.

**Infant factors** included gender, gestational age, skin-to-skin contact in the first 24 hours and early pacifier use.

**Mother-infant interaction factors** included four different scales validated in mothers of infants 0–12 months of age. The Karitane Parenting Confidence scale (KPCS) consisting of 15 questions that measured parenting confidence on a four-point Likert scale [29]. The Mother and Baby Interaction Scale (MABISC) consisting of 10 questions that measured maternal perception of mother-infant interaction on a five-point Likert scale [30]. The Ages and Stages questionnaire (ASQ:SE) consisting of 16 questions that measured parental perception of the infants´ social and emotional competences following birth on a four-point Likert scale [31]. The Major Depression Inventory (MDI) scale consisting of 10 questions that measured maternal mood and depression tendency on a six-point Likert scale [32].

**Psychosocial factors** included five questions available in content and wording from Kronborg & Vaeth [18]. Intention measured how long mothers planned to breastfeed exclusively. Outcome evaluation measured the value mothers attributed to successful breastfeeding. Self-efficacy measured maternal confidence in being able to breastfeed exclusively for four months. Social influence / subjective norms measured maternal perception of the degree to which influential people in her life encouraged her breastfeeding. Sense of security measured maternal coping with not knowing the exact amount of milk the newborn ingested at the breast. All variables except intention (measured in months) were measured on five-point Likert scales and dichotomized into high (1–2 points), moderate or low level (3–5 points).

### Statistical methods

Initially, we described the characteristics of the included mother-infant dyads stratified on parity, followed by a crude Cox proportional hazards analysis investigating the associations of each of the study variables with the outcome variable on exclusive breastfeeding duration.

**Fig 1:**
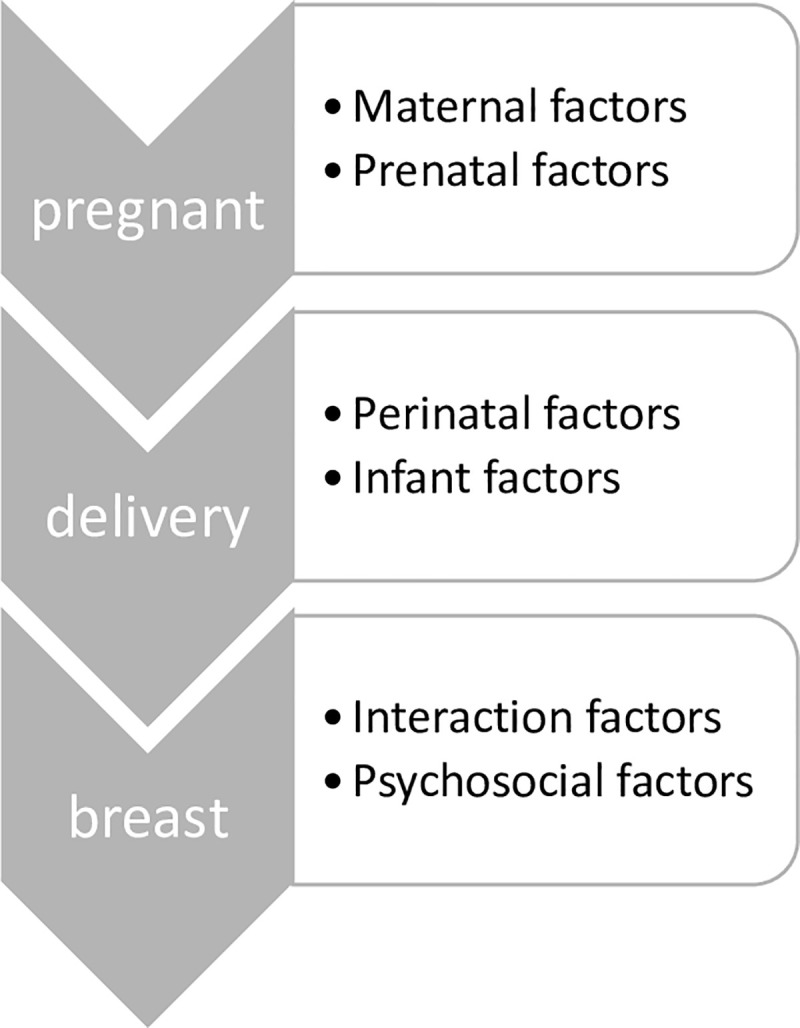
Conceptual model illustrating how different factors over time influence the duration of exclusive breastfeeding.

Next, we used multiple Cox proportional hazards models to assess the influence of the study variables on breastfeeding duration. Five models were estimated using a stepwise approach where the allocated groups of study variables were included one by one. We understood breastfeeding as a dynamic process influenced by events following time. Study variables were included in the stepwise model in accordance with the conceptualized model presented in Fig 1 with sociodemographic characteristics as the most distal factors (model 1), pre/perinatal-, infant-, and interaction-related factors as more proximal (models 2, 3, and 4), and finally psychosocial factors (model 5). Although the latter is formed early and cover the time spectrum, psychosocial factors may change over time depending on other factors [33]. In all models, we adjusted for allocation to the trial intervention.

The associations were presented as Hazard ratios (HR) describing the ratio for the cessation rate of exclusive breastfeeding for the given level of the study variable relative to the cessation rate for the reference level. A hazard ratio (HR) estimate is an average effect over time, but the effect of the study variables is not necessarily the same over time (non-proportional). Therefore, in additional analyses of the final model (model 5), all study variables were investigated for time-dependency by including interactions between the variables and log time in weeks. Study variables with time- varying effect were further assessed using Kaplan Meier survival curves.

## Results

A total of 3504 mothers gave birth in the four study municipalities during the study period. Of these, 2565 (73%) consented to participate in the study. Mothers who did not fill out the first questionnaire (n = 1442), who had never started breastfeeding (n = 66), and who lacked providing information on breastfeeding duration in the second or third questionnaire (n = 893) were excluded. Thus, the study population consisted of 1265 (49%) mothers who had completed questionnaire 1, started breastfeeding after birth, and provided valid information on duration of exclusive breastfeeding in the first or second follow-up questionnaire. Missing information on the study variables was generally low (0–2%), but higher for the four scales measuring mother-infant interaction (3–4%) and the measure of exclusive breastfeeding intention (5%).

Table 1 shows descriptive statistics for the study variables stratified on parity and crude associations with duration of exclusive breastfeeding. The study population consisted of 591 (47%) first-time mothers and 674 (53%) multiparous women. Mothers exclusively breastfed their infant for 15 weeks on average, and multiparous women around 1½ weeks longer than first-time mothers. At four months (17 weeks) and six months (26 weeks) postpartum, 340 (50%) and 666 (99%) multiparous women and 357 (60%) and 582 (98%) first-time mothers had stopped exclusive breastfeeding. Sociodemographic factors showed that 35% of mothers had a shorter or no vocational education, and 23% and 16%, respectively were overweight or obese. Pre- and peri-natal factors showed that 62% of the study population was hospitalized more than 24 hours following birth. Mothers assessed early information from health professionals as positive with an average score of 8.67 (range 0–25, lower scores were favorable). Nearly all mothers (95%) reported to have had at least one type of early physical breastfeeding problem. Infant-related characteristics showed that half of the infants in the study population were boys, and 40% of the infants had had less than five hours of skin-to-skin contact during the first 24 hours. Concerning mother-infant interaction, 285 (23%) mothers reported they had low maternal confidence (KPCS score below 40), and 151 (12%) mothers tended to be depressed with a MDI score above 12. Psychosocial factors showed that mothers had an intention of breastfeeding for an average of 4.5 months and more than three quarters of the mothers attributed positive value to breastfeeding, thus suggesting that breastfeeding was important to them. Only 13% of the mothers evaluated breastfeeding not to be important to them. At the same time one third of the mothers expressed low self-efficacy to succeed in breastfeeding (for four months) and more than a third expressed a low sense of security not knowing the exact amount of milk their infant ingested when being breastfed. When evaluated separately, all the mentioned study variables were significantly associated with the duration of exclusive breastfeeding in crude Cox regression models (Table 1).

**Table 1:** Characteristics of 1265 mother-infant dyads according to maternal, pre/perinatal, infant, bonding, and psychosocial factors by parity and crude associations with duration of exclusive breastfeeding.

Characteristics	Value	First-time mothers	Multiparous women	Total	
		n (%)	n (%)	n (%)	HR (95% CI)
**Outcome**					
Exclusive breastfeeding duration in weeks	*Mean (SD)*	*13*.*67 (8*.*51)*	*15*.*49 (7*.*93)*	*14*.*64 (8*.*25)*	
**Maternal factors**					
Age in years	*Mean (SD)*	*28*.*87 (4*.*28)*	*32*.*53 (4*.*01)*	*30*.*82 (4*.*52)*	0.99 (0.98–1.00)
Completed vocational education	Bachelor—master	358 (60.58)	461 (68.40)	819 (64.74)	Ref.
	None–short–skilled	233 (39.42)	213 (31.60)	446 (35.26)	**1.24 (1.11–1.39)**
BMI	Under- or normal weight	355 (60.07)	415 (61.57)	770 (60.87)	Ref.
	Overweight	150 (25.38)	143 (21.22)	293 (23.16)	1.09 (0.96–1.25)
	Obese	86 (14.55)	116 (17.21)	202 (15.97)	**1.27 (1.09–1.48)**
Smoking	No	557 (94.25)	644 (95.55)	1201 (94.94)	Ref.
	Yes	34 (5.75)	30 (4.45)	64 (5.06)	**1.36 (1.05–1.74)**
**Pre/perinatal factors**					
Parity	Multiparous women			674 (53.28)	Ref.
	First-time mothers			591 (46.72)	**1.17 (1.05–1.31)**
Breastfeeding duration previous child	17+ weeks		347 (52.42)	347 (27.69)	Ref.
	6–17 weeks		171 (25.83)	171 (13.65)	**1.76 (1.46–2.11)**
	0–5 weeks		144 (21.75)	144 (11.49)	**2.56 (2.10–3.11)**
	None, first-time mothers			591 (47.17)	**1.56 (1.37–1.78)**
Birth information, delivery	Vaginal birth	483 (81.73)	586 (86.94)	1069 (84.51)	Ref.
	Cesarean section	108 (18.27)	88 (13.06)	196 (15.49)	1.11 (0.96–1.30)
Birth information, hospitalization	Home birth—outpatient	104 (17.66)	381 (56.78)	485 (38.49)	Ref.
	Hospitalized > 24 hours	485 (82.34)	290 (43.22)	775 (61.51)	**1.17 (1.05–1.32)**
Early information from health professionals, index range 0–25*	*Mean (SD)*	*9*.*50 (3*.*51)*	*7*.*93 (3*.*77)*	*8*.*67 (3*.*73)*	**1.02 (1.00–1.03)**
Early physical breastfeeding problems, index range 0–12*	*Mean (SD)*	*3*.*87 (2*.*16)*	*3*.*79 (2*.*30)*	*3*.*83 (2*.*23)*	**1.02 (1.00–1.05)**
**Infant factors**					
Sex	Girl	291 (49.24)	346 (51.34)	637 (50.36)	Ref.
	Boy	300 (50.76)	328 (48.66)	628 (49.65)	**1.15 (1.03–1.28)**
Gestational age	> 37 weeks	564 (95.43)	663 (98.37)	1227 (97.00)	Ref.
	≤ 37 weeks	27 (4.57)	11 (1.63)	38 (3.00)	1.02 (0.73–1.41)
Skin-to-skin contact within first 24 hours	> 5 hours	378 (63.96)	389 (57.72)	767 (60.63)	Ref.
	≤ 5 hours	213 (36.04)	285 (42.28)	498 (39.37)	**1.15 (1.02–1.28)**
Pacifier use	No	124 (21.42)	132 (19.91)	256 (20.61)	Ref.
	Yes	455 (78.58)	531 (80.09)	986 (79.39)	1.22 (1.07–1.40)
**Maternal infant interaction factors**					
Maternal confidence, KPCS scale range 0–45**	*Mean (SD)*	*40*.*20 (3*.*86)*	*42*.*17 (2*.*95)*	*41*.*25 (3*.*54)*	**0.98 (0.96–0.99)**
Mother-infant interaction, MABISCH scale range 0–40*	*Mean (SD)*	*9*.*30 (4*.*48)*	*7*.*99 (4*.*07)*	*8*.*60 (4*.*31)*	1.00 (0.99–1.02)
Maternal mood and depression tendency, MDI scale range 0–50*	*Mean (SD)*	*7*.*85 (5*.*93)*	*6*.*09 (4*.*83)*	*6*.*91 (5*.*44)*	**1.02 (1.01–1.03)**
Infant social and emotional competencies, ASQ:SE scale range 0–240*	*Mean (SD)*	*28*.*37 (15*.*17)*	*24*.*83 (13*.*26)*	*26*.*49 (14*.*29)*	1.00 (1.00–1.00)
**Psychosocial factors**					
Exclusive breastfeeding intention, months	*Mean (SD)*	*4*.*56 (2*.*10)*	*4*.*46 (1*.*93)*	*4*.*51 (2*.*01)*	**0.70 (0.67–0.72)**
Outcome evaluation of breastfeeding	Important	507 (87.56)	576 (86.88)	1083 (87.20)	Ref.
	Not important	72 (12.44)	87 (13.12)	159 (12.80)	**2.61 (2.20–3.09)**
Self-efficacy	Certain	368 (63.56)	462 (69.68)	830 (66.83)	Ref.
	Uncertain	211 (36.44)	201 (30.32)	412 (33.17)	**2.47 (2.19–2.79)**
Social influence, subjective norm	Positive	317 (54.75)	364 (54.98)	681 (54.88)	Ref.
	Negative	262 (45.25)	298 (45.02)	560 (45.12)	**1.35 (1.21–1.51)**
Sense of security not knowing amount of milk ingested	Secure	267 (46.11)	436 (65.76)	703 (56.60)	Ref.
	Insecure	312 (53.89)	227 (34.24)	539 (43.40)	**1.79 (1.60–2.00)**

Coefficients in bold are significant at a 5% significant level; Missing values excluded.

*Scale low scores are favorable

** scale high scores favorable

The results from the multiple stepwise Cox regression analysis are shown in Table 2. The following factors showed an independent statistically significant association with a shorter exclusive breastfeeding duration in the final model. Among pre/perinatal factors, duration of breastfeeding in the previous child was significantly associated with current breastfeeding duration. Compared to multiparous women who had breastfed their previous infant for more than 17 weeks, multiparous women who had breastfed for five weeks and first-time mothers who had never breastfed had an increased cessation rate of 45% and 24%, respectively (HR 1.45, CI: 1.15–1.82; HR 1.24, CI: 1.04–1.49). Among infant factors, skin-to-skin contact for five hours or less during the first 24 hours of life was associated with an increased cessation rate of 18%, compared to more skin-to-skin contact (HR 1.18, CI: 1.04–1.33). Among mother-infant interaction factors, low maternal confidence (KPCS scale) and mother-infant interaction (MABISCH scale) had marginal or no association with breastfeeding duration (HR: 0.97, CI: 0.95–1.00; HR: 0.97, CI: 0.95–0.99, respectively). Among psychosocial factors, an expressed intention to breastfeed for a longer period predicted a longer breastfeeding duration with a decrease in the cessation rate of 33% per month of extra intended breastfeeding duration (HR 0.77, CI: 0.73–0.80). Moreover, evaluating the outcome of breastfeeding to be low, expressing a low self-efficacy towards being able to breastfeed for four months, and insecurity about not knowing the amount of milk the infant ingested when being breastfed were associated with increased cessation rates (HR: 1.33, CI: 1.08–1.63; HR 1.46, CI: 1.24–1.72; HR: 1.31, CI: 1.14–1.50, respectively). None of the sociodemographic factors showed an independent prognostic effect when adjusting for the other factors included in the final model. A significant association between maternal obesity and breastfeeding duration disappeared when the pre/perinatal factors were added to the model (Model 2); an association with educational level was statistically insignificant when adjusting for the psychosocial factors (Model 5).

**Table 2:** Cox proportional hazards models for associations between study variables and exclusive breastfeeding duration, n = 1156.

Characteristics	Model 1	Model 2	Model 3	Model 4	Model 5
	HR (95% CI)	HR (95% CI)	HR (95% CI)	HR (95% CI)	HR (95% CI)
**Sociodemographic factors**					
Age in years	1.00 (0.98–1.01)	1.01 (0.99–1.03)	1.01 (0.99–1.03)	1.01 (1.00–1.03)	1.00 (0.99–1.02)
Educational level: Skilled, short or no education	**1.19 (1.04–1.35)**	**1.16 (1.01–1.32)**	**1.14 (1.00–1.31)**	**1.15 (1.00–1.31)**	1.04 (0.91–1.19)
BMI					
Underweight or normal	Ref.	Ref.	Ref.	Ref.	Ref.
Overweight	1.05 (0.91–1.21)	1.04 (0.90–1.19)	1.04 (0.90–1.20)	1.05 (0.91–1.22)	1.01 (0.87–1.17)
Obese	**1.20 (1.20–1.41)**	1.07 (0.90–1.27)	1.06 (0.90–1.26)	1.06 (0.89–1.26)	0.97 (0.82–1.16)
Smoker	1.22 (0.94–1.60)	1.11 (0.84–1.46)	1.10 (0.84–1.45)	1.08 (0.82–1.43)	0.95 (0.72–1.25)
**Pre/perinatal factors**					
Breastfeeding duration previous child					
17+ weeks		Ref.	Ref.	Ref.	Ref.
6–17 weeks		**1.76 (1.45–2.13)**	**1.75 (1.45–2.12)**	**1.73 (1.43–2.09)**	1.21 (0.99–1.47)
0–5 weeks		**2.54 (2.05–3.15)**	**2.52 (2.04–3.13)**	**2.54 (2.05–3.15)**	**1.45 (1.15–1.82)**
None, first-time mothers		**1.61 (1.35–1.91)**	**1.61 (1.35–1.92)**	**1.58 (1.33–1.89)**	**1.24 (1.04–1.49)**
Cesarean section		1.06 (0.89–1.26)	1.05 (0.88–1.24)	1.04 (0.87–1.23)	0.99 (0.83–1.18)
Hospitalized > 24 hours following birth		0.99 (0.86–1.15)	1.00 (0.87–1.15)	1.01 (0.87–1.16)	1.05 (0.91–1.21)
Early information from health professionals*		1.02 (1.00–1.03)	1.01 (1.00–1.03)	1.01 (1.00–1.03)	1.00 (0.98–1.02)
Early physical breastfeeding problems*		1.01 (0.98–1.04)	1.01 (0.99–1.04)	1.01 (0.99–1.04)	1.01 (0.98–1.04)
**Infant factors**					
Boy			1.10 (0.97–1.23)	1.10 (0.97–1.23)	1.06 (0.94–1.20)
Gestational age: > 37 weeks			1.09 (0.76–1.55)	1.08 (0.75–1.56)	1.06 (0.74–1.51)
Skin-to-skin contact within first 24 hours: ≤ 5 hours			**1.15 (1.02–1.30)**	**1.15 (1.02–1.30)**	**1.18 (1.04–1.33)**
Pacifier use			**1.17 (1.01–1.35)**	1.15 (1.00–1.33)	1.01 (0.87–1.17)
**Mother-infant interaction factors**					
Maternal confidence, KPCS scale**				**0.97 (0.95–0.99)**	**0.97 (0.95–1.00)**
Mother-infant interaction, MABISCH scale*				**0.98 (0.96–1.00)**	**0.97 (0.95–0.99)**
Maternal mood and depression tendency, MDI scale*				1.01 (0.99–1.02)	1.01 (0.99–1.02)
Infant social and emotional competencies, ASQ:SE scale*				1.00 (0.99–1.00)	1.00 (0.99–1.00)
**Psychosocial factors**					
Exclusive breastfeeding intention, months					**0.77 (0.73–0.80)**
Outcome evaluation: Important					**1.33 (1.08–1.63)**
Self-efficacy: Certain					**1.46 (1.24–1.72)**
Social influence, subjective norm: Positive					1.08 (0.95–1.22)
Sense of security not knowing amount of milk ingested: Secure					**1.31 (1.14–1.50)**

Coefficients in bold are significant at a 5% significant level; Adjustment for intervention group was included in all models

*Scale low scores favorable

** scale high scores favorable

**Fig 2:**
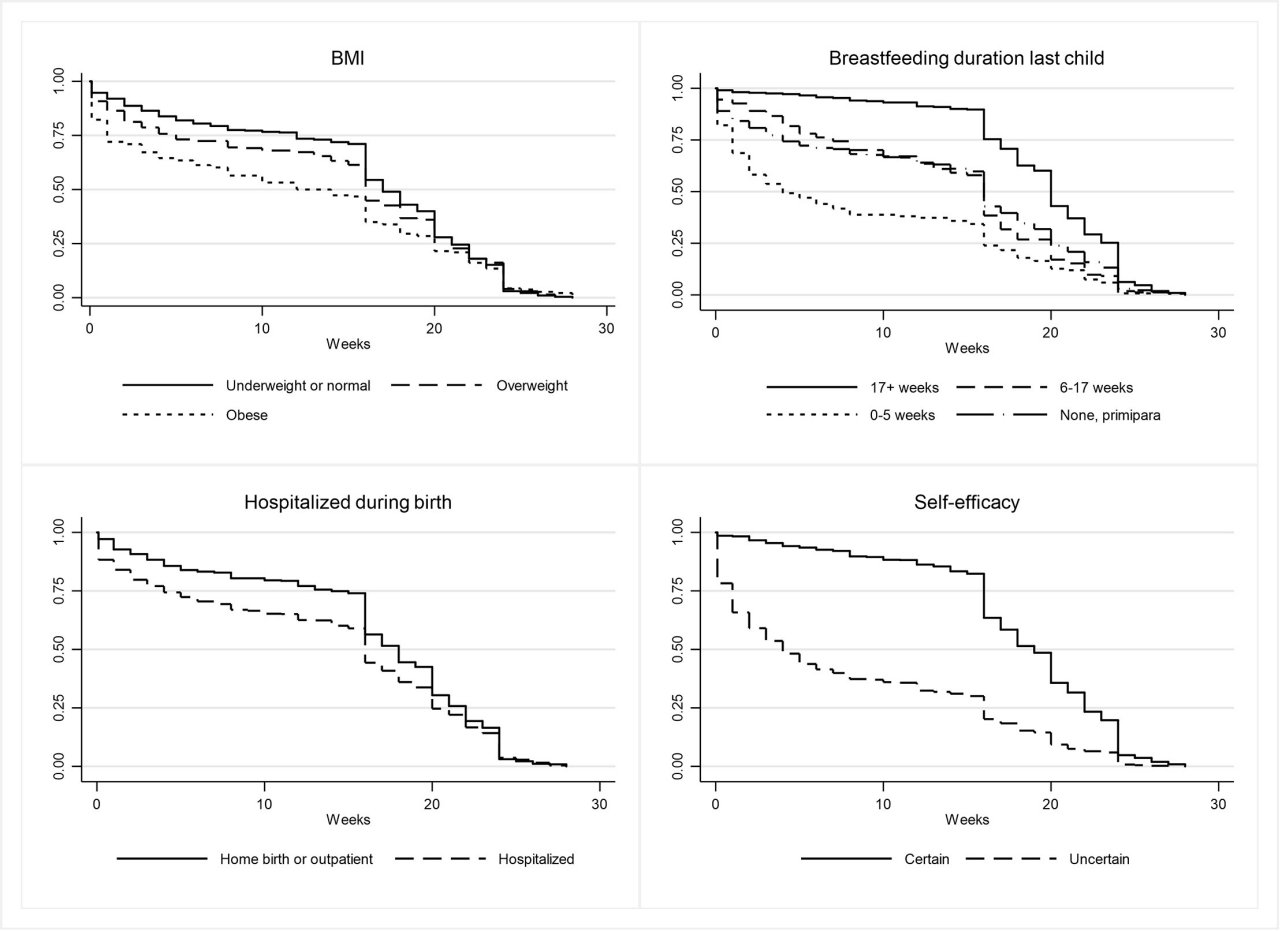
Kaplan Meier curves showing the probability (percentage) of mothers exclusively breastfeeding by time for the four factors BMI, breastfeeding duration of previous child, hospitalization during birth and self-efficacy, n = 1156.

In additional analyses, we examined if the influence of the study variables varied with time in the final model (Model 5). Results (S1 Table) showed that the association of four of the study variables was dependent on time. These included BMI categories among the sociodemographic factors, breastfeeding duration of the previous child, hospitalization during birth among the pre/perinatal factors and finally self-efficacy among the psychosocial factors. Fig 2 shows the proportion of mothers exclusively breastfeeding as a function of time by these four variables. In general, the difference in probability of breastfeeding between the groups was more extensive (had more influence) in the first months following birth and decreased over time. For example, obese mothers had a higher cessation rate compared to underweight and normal weight mothers during the first 16 weeks after birth; after this time point, the difference between the two groups narrowed markedly.

## Discussion

The results of this study confirmed the multifactorial structure of breastfeeding with a number of factors independently associated with a longer duration of exclusive breastfeeding. These factors included early skin-to skin contact, maternal intention to breastfeed, positive outcome evaluation describing the value mothers attributed to breastfeeding, self-efficacy, and maternal sense of security in relation to breastfeeding. The factors of higher maternal BMI, shorter breastfeeding duration of previous child, and being hospitalized during birth were found to be time-dependent and only influenced breastfeeding duration in the first months postpartum. Finally, an association with a shorter breastfeeding duration was initially seen among mothers with a lower level of education, but in the fully adjusted model, educational level had no independent prognostic effect.

Strengths of the study included data covering a diverse population of mothers, access to data on all currently known risk factors influencing breastfeeding duration, and the cohort design ensuring a stepwise collection of data limiting recall bias. Not included or unknown factors with significant influence may, however, increase the risk of bias. For example, we did not have data on milk expression practices which have been suggested to decrease breastfeeding duration [34]. Additional limitations included the high attrition rate leading to an analytical sample only including 49% of the original population invited to participate. However, comparing the analytical sample to the general population we found that the analytical sample of mothers was representative of the Danish background population according to age, level of education and ethnicity. Yet, a small underrepresentation of mothers who had given birth to a premature infant was seen, and we cannot rule out that results are affected by selection bias. Breastfeeding duration as all other data was self-reported. Even though mothers usually recall their breastfeeding duration quite well [35], it may have been more reliable if data on the outcome variable of exclusive breastfeeding had been collected by health professionals. We addressed possible incorrect information by cross checking the time stated for breastfeeding cessation with the time stated for initiation of giving food other than breast milk. This correction may be the reason why the proportions of exclusive breastfeeding duration measured in the present study is a little lower than usually measured in a Danish population of new mothers [12,18].

Psychosocial factors dominated among the variables associated with exclusive breastfeeding. As most women who become mothers are biologically able to breastfeed, the choice of breastfeeding in western societies may be perceived as a choice of health behavior. Intention measured the maternal goal for breastfeeding, the self-efficacy component expressed the expectations to be able to accomplish the task, and the positive outcome evaluation measured the value mothers attributed to breastfeeding [18]. According to theory by Albert Bandura, the thoughts of a positive outcome provide positive motivation and incentives to perform the desired health behavior. Yet, similar to the results of the present study, the self-efficacy component is a more powerful factor for obtaining prognostic information on breastfeeding duration, probably because it predicts a mother's self-efficacy beliefs and thereby her effort to overcome potential problems [36]. A low level of self-efficacy is more likely to leave the mother confused with the urge to give up if unexpected problems occur early, as is often the case in breastfeeding. This may explain why early breastfeeding problems were not found to be predictive of duration of exclusive breastfeeding in this study. It was rather the mother's level of self-efficacy that was crucial for her confidence in overcoming early breastfeeding problems and continuing to breastfeed. Sense of security not knowing the amount of milk ingested reflected the insecurity that mothers may feel when they are new and inexperienced. Breastfeeding is an infant-led approach because the infant controls the milk intake. In order to gain a sense of security towards breastfeeding, the breastfeeding mother has to cope with a more responsive feeding style and learn to read the infants cues of satiety and hunger to gain knowledge of the amount of milk the infant ingests [37,38]. The influence of psychosocial determinants on breastfeeding duration has been confirmed by a number of studies [18–20,33]. The importance of having skin-to skin contact with your baby during the first 24 hours after birth and its positive effect on exclusive breastfeeding duration has also been shown earlier [17]. The importance of this may be due to its effect on the onset of breastfeeding through both the child's active search for the breast and the mother's sensitive behavior towards the child [26,39]. The representation of independent associations with factors from different domains and breastfeeding duration underlined the multifactorial structure of breastfeeding and the necessity to include knowledge of psychosocial factors if breastfeeding support is going to have an effect. According to Bandura [35], sources to increase self-efficacy should first and foremost focus on giving the mother positive experiences with being able to master the task. Here, the health professional can help by agreeing on small sub-goals with the mother and applying observational learning. Each time a small sub-goal is met, the mother will experience her confidence in breastfeeding increase. In addition, verbal persuasion, which convinces the mother that she is able to accomplish the task is important. The vulnerable mother who wants to breastfeed must not be left in doubt, but instead receive practical guidance and positive feedback to succeed. Making use of knowledge about the psychosocial factors in breastfeeding support has shown positive effects on breastfeeding duration [40], but more research and intervention studies in this area are required.

The present study showed that if mothers with characteristics of being obese, hospitalized during birth, or having low levels of experience concerning breastfeeding had managed the first months and established breastfeeding, they were more or less just as likely to continue exclusive breastfeeding for as long as mothers without these characteristics. Hospitalization, obesity, and low levels of experience or self-efficacy concerning breastfeeding have previously been demonstrated to be associated with an increased risk of breastfeeding cessation in the first vulnerable period [41,42,18]. Being hospitalized during childbirth indicates a potentially greater need for follow-up by healthcare professionals [41]. Maternal obesity includes increased difficulty in starting breastfeeding due to hormonal and mechanical factors [42]. Having previous short breastfeeding experience tend to repeat practices from the previous infant [12], and a low level of self-efficacy results in less confidence in overcoming early breastfeeding problems [20]. This study importantly pointed out that these factors had varying influence over time by being most influential in the first months following birth. This suggests that mothers who had been hospitalized during birth, obese mothers, mother with low self-efficacy towards being able to succeed in breastfeeding, and multiparous mothers who have only breastfed their previous child for a short period are specific target groups with special needs for early supplemental support to establish breastfeeding [39].

Similar to other studies [5,6,43], the present study found that maternal level of education was associated with breastfeeding duration. The association, however, became weaker when adjusting for more proximal factors influencing breastfeeding duration, especially when considering psychosocial factors. This suggests that a possible influence of educational background on breastfeeding duration might be explained by psychosocial factors; however, more sophisticated mediation analyses would be needed to fully test this hypothesis. The current study was conducted in Denmark with a relatively well-educated population and a strong breastfeeding tradition. Since the measurement of breastfeeding determinants has not proved to be considerably different across countries, there is reason to believe that our results can be generalized to other western societies.

## Conclusions

In a study that supports and extends the concept of breastfeeding as a multifactorial health behavior, we found that a number of different factors had an independent and long-term prognostic influence on duration of exclusive breastfeeding. These factors included early skin-to-skin contact, intention to breastfeed, level of self-efficacy, and sense of security as well as the value mothers attributed to breastfeeding. Other factors showed a time dependency by primarily affecting the exclusive breastfeeding duration in the first months following birth. These included hospitalization in connection to birth, maternal obesity and low self-efficacy as well as a short previous breastfeeding experience. These characteristics pointed to target groups in special need of early breastfeeding support, as the characteristics specifically influenced breastfeeding in the first months and were no longer important if the mother managed to successfully establish breastfeeding of her infant. The dominating influence of psychosocial aspects among the factors with an independent and long- term influence on exclusive breastfeeding duration underlined the importance of including psychosocial aspects in effective breastfeeding support. Not only as prognostic factors to identify mothers in need of extra support, but also to include psychosocial aspects in the practical guidance helping the mother to gain positive breastfeeding experiences. Practices may include modelling and observational learning to help the mother master sub-skills of breastfeeding as well as addressing maternal uncertainties verbally and convincing her of her ability to successfully breastfeed.

## Supporting information

S1 Table Cox proportional hazards model for associations between study variables and exclusive breastfeeding including significant interactions between study variables and log time in weeks, n = 1156. (DOCX)
