# Supplementary material for: Multifactorial influence on duration of exclusive breastfeeding; a Danish cohort study
Source: PLoS One. 2020 Sep 1;15(9):e0238363. doi: 10.1371/journal.pone.0238363 (PMC7462295; doi:10.1371/journal.pone.0238363)
Supplement: S1 Table — (DOCX) [file pone.0238363.s001.docx]

**S1 Table: Cox proportional hazards model for associations between study variables and exclusive breastfeeding including significant interactions between study variables and log time in weeks, n = 1156**

| **Characteristic** | Model 5 | |
| --- | --- | --- |
|  | HR | 95% CI |
| **Maternal factors** |  |  |
| Age in years | 1.00 | 0.98, 1.02 |
| Educational level: None, short or skilled | 1.06 | 0.93, 1.22 |
| BMI |  |  |
| Underweight or normal | Ref. |  |
| Overweight | 1.21 | 0.95, 1.56 |
| Obese | 1.24 | 0.97, 1.59 |
| Smoker | 0.91 | 0.69, 1.20 |
| **Pre- and perinatal factors** |  |  |
| Breastfeeding duration last child |  |  |
| 17+ weeks | Ref. |  |
| 6-17 weeks | 3.46 | 1.65, 7.25 |
| 0-5 weeks | 5.01 | 2.44, 10.29 |
| Non, first-time mothers | 4.42 | 2.18, 8.94 |
| Cesarean | 0.98 | 0.82, 1.17 |
| Hospitalized > 24 hours following birth | 1.47 | 1.13, 1.91 |
| Early information from health professionals* | 1.00 | 0.98, 1.02 |
| Early physical breastfeeding problems* | 1.00 | 0.97, 1.03 |
| **Infant factors** |  |  |
| Boy | 1.06 | 0.94, 1.19 |
| Gestational age: > 37 weeks | 0.96 | 0.67, 1.37 |
| Skin to skin first 24 hours: ≤ 5 hours | 1.18 | 1.05, 1.34 |
| Use pacifier | 1.06 | 0.92, 1.23 |
| **Mother infant interaction factors** |  |  |
| Maternal confidence, KPCS scale** | 0.98 | 0.96, 1.00 |
| Mother baby interaction, MABISCH scale* | 0.98 | 0.96, 1.00 |
| Maternal mood and depression tendency, MDI scale* | 1.00 | 0.99, 1.02 |
| Infant social and emotional competences, ASQ:SE scale* | 1.00 | 0.99, 1.00 |
| **Psychosocial factors** |  |  |
| Exclusive breastfeeding intention, months | 0.81 | 0.77, 0.84 |
| Outcome evaluation: Important | 1.22 | 0.99, 1.50 |
| Self-efficacy: Certain | 4.35 | 2.96, 6.39 |
| Social influence, subjective norm: Positive | 1.06 | 0.94, 1.20 |
| Sense of security not knowing milk ingest: Secure | 1.30 | 1.13, 1.49 |
| **Interactions with log time in weeks** |  |  |
| BMI |  |  |
| Underweight or normal | Ref. |  |
| Overweight | 0.92 | 0.84, 1.01 |
| Obese | 0.87 | 0.79, 0.95 |
| Breastfeeding duration last child |  |  |
| 17+ weeks | Ref. |  |
| 6-17 weeks | 0.72 | 0.55, 0.93 |
| 0-5 weeks | 0.65 | 0.50, 0.84 |
| Non, first-time mothers | 0.64 | 0.50, 0.81 |
| Hospitalized > 24 hours following birth | 0.86 | 0.78, 0.95 |
| Self-efficacy: Certain | 0.62 | 0.53, 0.71 |

Notes: Adjusted for intervention group

*Scale low scores favorable, ** scale high scores favorable
